# Effects of lavender oil preparation silexan on different symptoms of major depression – results from a randomized, controlled trial

**DOI:** 10.1093/ijnp/pyag041

**Published:** 2026-08-04

**Authors:** Hans-Jürgen Möller, Ion-George Anghelescu, Hans-Peter Volz, Erich Seifritz, Sandra Schläfke, Siegfried Kasper

**Affiliations:** Department of Psychiatry and Psychotherapy, Ludwig Maximilian University, Munich, Germany; Clinic of Psychiatry and Psychotherapy, Mental Health Institute Berlin, Berlin, Germany; Former Medical Director Hospital for Psychiatry, Psychotherapy and Psychosomatic Medicine Schloss Werneck, Werneck, Germany; Department of Psychiatry, Psychotherapy and Psychosomatics, Psychiatric Hospital, University of Zürich, Munich, Switzerland; Research & Development, Dr. Willmar Schwabe GmbH & Co. KG, Karlsruhe, Germany; Department of Psychiatry and Psychotherapy, Ludwig Maximilian University, Munich, Germany; Center for Brain Research, Medical University of Vienna, Vienna, Austria

**Keywords:** silexan, lavender, depression, clinical trial, treatment efficacy

## Abstract

**Background:**

Major depressive disorder (MDD) is characterized by depressed mood, anhedonia, and loss of energy, which can be accompanied by associated symptoms and co-morbidities. Psychiatric scales such as the Montgomery Åsberg Depression Rating Scale (MADRS) must account for the complex nature of depression.

**Methods:**

The MADRS total score change between baseline and week 8 was the primary outcome measure in a randomized, double-blind clinical trial investigating the antidepressant efficacy of 8 weeks’ treatment with silexan compared to sertraline and placebo in patients with mild or moderate MDD. We report on a pre-planned, exploratory analysis of the individual MADRS items. Treatment effects were assessed using analyses of covariance with baseline adjustment, based on an estimand strategy.

**Results:**

498 subjects (silexan 170, sertraline 171, placebo 157) were treated and analyzed. After 8 weeks, silexan was superior to placebo for 5 out of the 9 MADRS items analyzed (“apparent sadness”, “reported sadness”, “reduced appetite”, “concentration difficulties”, “lassitude”; *P* < .05) and showed clinically important adjusted mean value differences >0.2 points for 7 out of the 9 items. Item-level results for silexan and sertraline were mainly comparable.

**Conclusions:**

Silexan had a strong over-all antidepressant effect, with the most pronounced improvements affecting the cardinal symptoms of depression.

**Trial registration:**

EudraCT2020-000688-22 first entered on 12/08/2020.

**Significance statement**

Patients with depressive disorders can show many different symptoms. To better characterize the clinical action of an antidepressant, it is therefore important to analyze not only the overall value of a depression scale but also the individual items that describe these symptoms. Silexan is a preparation from lavender oil whose antidepressant effect has been proven in a randomized, double-blind, placebo-controlled 8-week study in patients with mild or moderate major depressive disorder. Based on the individual items of the Montgomery Åsberg Depression Rating Scale that was used as the main outcome for efficacy, we found in an exploratory, hypothesis-generating analysis that silexan had a rather broad antidepressant effect in the participants of our study, with potentially clinically meaningful advantages over placebo for 7 out of the 9 individual items investigated. This applied in particular to the main symptoms of depression, namely sadness and lassitude. Our single-item analysis thus helps to understand the antidepressant effects of silexan in more detail.

**Significant outcomes**

- In patients with mild to moderate major depressive disorder, lavender oil preparation silexan has a clinical profile similar to that of the selective serotonin re-uptake inhibitor sertraline based on an item-level analysis of the Montgomery-Åsberg Depression Rating Scale (MADRS).
- Silexan has a significant antidepressant effect that includes an alleviation of depressed mood, anhedonia, and loss of energy, the cardinal symptoms of depression. The broad improvement of symptoms of depression could not be explained by the proven anxiolytic efficacy silexan alone but indicates an independent, direct antidepressant effect.
- The present item-level analysis provides valuable and detailed additional insights into the therapeutic profiles of silexan and sertraline and may help clinicians to tailor antidepressant treatment to the specific symptoms of a patient.

**Limitations**

- For item-level analyses of the MADRS, no validated thresholds for the assessment of the clinical importance of changes over time have been defined, taking into account that different items may have different thresholds.
- Even though our analyses were pre-defined, they were exploratory and did not include studywise type I error level control. Their generalizability beyond the study population is therefore limited.

## Graphical Abstract

**Graphical Abstract:**
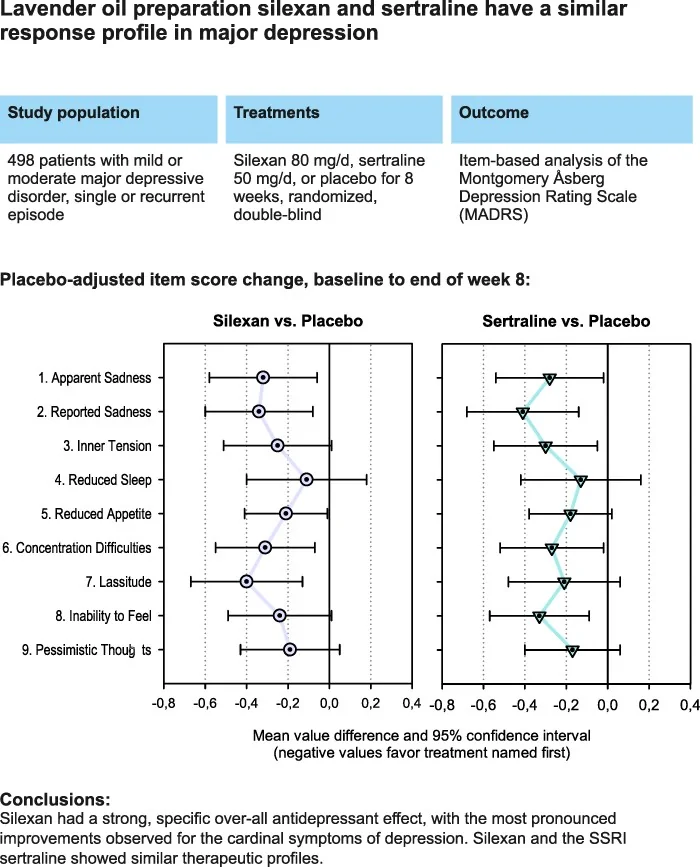

## Introduction

Depressive disorders are characterized by depressed mood, anhedonia, and loss of energy, which can be accompanied by loss of confidence or self-esteem, unreasonable feelings of self-reproach or guilt, impaired concentration, indecisiveness, psychomotor agitation or retardation, disturbed sleep, change in appetite, or suicidal ideation.^1^^,^^2^ Those symptoms vary in severity and presentation across individuals. Diagnostic tools used in everyday clinical practice and in therapy research must therefore account for the complex, heterogeneous nature of depression for obtaining a differentiated picture of the course of a depressive episode and optimizing treatment choices.

The clinician-rated Montgomery-Åsberg Depression Rating Scale (MADRS^3^) is a ten-item diagnostic instrument with total scores ranging from 0 to 60 points that is used extensively in every-day practice as well as in clinical research and assesses a broad range of symptoms associated with depression. Unlike the Hamilton Depression Rating Scale (HAMD^4^), the MADRS does not assess somatic symptoms and is thus robust against bias resulting from a confounding of bodily symptoms of depressions and adverse drug reactions.^3^ The scale was demonstrated to have favorable psychometric properties and to be sensitive to change.^3^^,^^5^ The latter was not only shown for the total score determined by summing up the item scores, but change of individual item scores was also found to predict clinical outcome.^6–8^

Conventionally, multi-item clinical outcome assessments (COA) are designed to cover a spectrum of symptoms or complaints that together allow a characterization of the condition whose severity the scale is designed to assess. In complex conditions such as depression, COA items may therefore be more or less heterogeneous in order to enable an adequate characterization of the clinical picture. For the MADRS, three factor analyses performed independently on different data sets resulted in solutions with four factors interpreted as “sadness”, “negative thoughts”, “detachment”, and “neurovegetative symptoms”,^9–11^ thus reflecting the heterogeneous nature of major depression.

While the MADRS total score of a scale has been validated as an indicator of the over-all severity of depression,^3^^,^^5^ the clinical manifestations assessed using the individual items may contribute differently to the overall burden, and patients with the same total score may have quite different individual item scores. The same applies to change over time, where changes in the total score may be driven by specific items rather than by uniform changes across all areas. Focusing the interpretation of the data on the total score alone may miss clinically important information when an improvement of the score is interpreted as a change in the construct as a whole while it may in fact have resulted from changes in a limited number of specific symptoms. Treatment effects may also vary across symptoms and may differ depending on the mechanism of action of the intervention.^12^

For example, an item-based analysis of the Hamilton Depression Rating Scale (HDRS^4^) using data from 32 clinical trials found that the apparently superior antidepressant effect of mirtazapine over selective serotonin reuptake inhibitors (SSRIs) in meta-analyses with the HDRS total score as the main outcome was attributable to the sedative and orexigenic properties of mirtazapine and its lack of gastro-intestinal side effects.^13^ Lisinski *et al.*^14^ used data from a total of 41 trials for indirect comparisons of the effects of duloxetine and several SSRIs based on item-level analyses and observed striking similarities between the HDRS item response profiles of the substances. For duloxetine, they found a robust beneficial effect on depressed mood already after 1 week, which was, however, masked by a concomitant deterioration in adverse event-related items when outcome was assessed based on the HDRS total score. Boschloo *et al.*^15^ meta-analyzed individual item data from trials comparing the efficacy of antidepressant medication and cognitive behavioral therapy (CBT) and found that advantages of drug treatment over CBT observed in some of the studies were mainly related to five specific symptoms represented by items of the HDRS. The authors concluded that information about symptom-specific efficacy could help to identify patients who, based on their pre-treatment symptomatology, are likely to benefit from a specific intervention. Vice versa, such information could also help to identify the intervention that might be the most promising for the treatment of a specific patient. In major depressive disorder (MDD), understanding the differential effects of treatments reflected in the individual items of psychiatric symptom scales may thus enable more precise therapeutic decision-making and the tailoring of treatments to specific patient needs.

Silexan [Silexan® is the active substance of Lasea® (Dr. Willmar Schwabe GmbH & Co. KG, Karlsruhe, Germany)] is an essential oil for oral administration produced from *Lavandula angustifolia* flowers that is registered as a medicinal product for the treatment of anxiety disorders. In randomized, placebo-controlled clinical trials in patients with anxiety, silexan consistently showed a significant beneficial effect on co-morbid depression.^16^ In a placebo-controlled study in mixed anxiety and depressive disorder in which the MADRS total score was a co-primary endpoint, a significant antidepressant effect was demonstrated for silexan.^17^ Moreover, the recently published, randomized, double-blind, double-dummy Lasea in depression (LEAD) study, the first trial with silexan in patients with mild or moderate MDD according to International Statistical Classification of Diseases and Related Health Problems (ICD)-10 criteria,^18^ demonstrated that treatment with 1 × 80 mg/d of the product for 8 weeks has an antidepressant effect superior to placebo and comparable to 1 × 50 mg/d of sertraline.^19^

Depression and anxiety disorders are highly comorbid^20^^,^^21^ and show overlapping symptoms including anhedonia, sad mood, and worry^22^ as well as common neurobiological alterations.^23^ Antidepressant drugs, including SSRIs and selective norepinephrine reuptake inhibitors (SNRIs), are also recommended as first line treatment for anxiety disorders.^24^

The antidepressant efficacy of silexan could be explained by a beneficial effect of lavender oil on neuroplasticity and neurogenesis,^25^^,^^26^ which is assumed to be a common pathway of antidepressant drugs in general.^27^ Moreover, silexan was found to increase the extracellular serotonin, dopamine, and norepinephrine levels,^28^^,^^29^ a mechanism typically observed in re-uptake inhibiting antidepressants like SSRIs. The antidepressant effect of silexan is thus consistent with its pharmacological profile.^30^

The primary efficacy outcome measure of the LEAD study^19^ was the absolute change of the MADRS total score between baseline and the end of week 8. To better understand the clinical manifestations of the antidepressant effect of silexan, we performed a pre-planned, exploratory analysis of the individual items of the MADRS to investigate differential changes in the severity of the symptoms of MDD between patients treated with silexan, sertraline, or placebo using the data of the LEAD study.

Given the high comorbidity anxiety and depressive disorders, it could be speculated that the efficacy of drugs in both conditions might be explained by their effect on overlapping symptoms. An item-based analysis like the one reported here could offer an opportunity to explore this.

## Methods

### Study characteristics

The LEAD study^19^ was a double-blind, double-dummy, randomized, placebo, and reference controlled parallel-group trial performed to evaluate antidepressant efficacy, safety, and tolerability of silexan. Adult out-patients with mild or moderate, single, or recurrent MDD according to ICD-10 categories F32.0, F32.1, F33.0, or F33.1^18^ were eligible for participation if they had a MADRS total score between 19 and 34 points at both the baseline and a screening visit performed 3–7 days earlier. Patients with severe MDD, any clinically important psychiatric (notably anxiety disorders) or neurological diagnosis other than the study indication within 6 months before enrolment, or who had received any psychotropic drugs, intravenous methylene blue, or linezolid within 30 days, or psychotherapy within 2 weeks before randomization, were excluded, as were those with a score of ≥1 point for the Beck Scale for Suicide Ideation 5-Item Screen^31^ or for MADRS item “Suicidal thoughts” at screening or baseline. Patients who had not responded to adequate antidepressant pharmacotherapy in the current depression episode, or who had failed to respond to a daily dose of ≥50 mg of sertraline for at least 6 weeks in any previous episode, were also not accepted.

Silexan is a proprietary essential oil produced from *L. angustifolia* complying with the monograph Lavender oil of the European Pharmacopeia (Ph. Eur.^32^). It exceeds the quality definition of the Ph. Eur. Eligible patients were randomized to receive 80 mg/d of silexan, 50 mg/d of sertraline, or placebo (ratio: 1:1:1 stratified by site) for 56 days, followed by double-blind down-titration of sertraline. The investigational products were to be taken once daily in the morning, under double-blind, double-dummy conditions (ie, silexan plus sertraline placebo, sertraline plus silexan placebo, or silexan placebo plus sertraline placebo).

Participants were assessed for treatment efficacy and safety at 1, 2, 4, 6, and 8 weeks after baseline. In accordance with the estimands framework of the trial, subjects terminating treatment prematurely were asked to continue participating in the study procedures until the scheduled end.

The study protocol was reviewed and approved by the independent ethical committees of the State Medical Association of Lower Saxony, Germany (process no. Bo/36/2020) and at the Regional Medical Chamber in Gdańsk, Poland (process no. 935 / 2022) as well as by the German and Polish competent authorities (EudraCT no. 2020-000688-22). All subjects provided written informed consent to participate and for the processing of their data. The principles of Good Clinical Practice and the Declaration of Helsinki were adhered to.

### MADRS assessments

The MADRS is a clinician-rated scale designed to assess the severity of depression; it has been demonstrated to be sensitive to change over time.^3^ The scale includes ten items, each scored from 0 (normal / absence of symptom) to 6 (symptom is severe / continuously present), that are summed up to a total score ranging between 0 and 60 points. Item 10 (“suicidal thoughts”) was not included in the analysis of individual MADRS items because participants eligible for randomization were required to have an item score of 0 at screening and baseline, and patients with a score >0 during randomized treatment had to be withdrawn from the trial in accordance with the investigational protocol. The remaining included items were: 1 – “apparent sadness”; 2 – “reported sadness”; 3 – “inner tension”; 4 – “reduced sleep”; 5 – “reduced appetite”; 6 – “concentration difficulties”; 7 – “lassitude”; 8 – “inability to feel”; 9 – “pessimistic thoughts”. Raters were psychiatrists or general practitioners. Uniformity of assessments was assured by performing a mandatory rater training (including a performance review with determination of the inter-rater reliability) before the start of patient recruitment.

The MADRS was assessed at baseline and at all post-baseline visits until the end of treatment. The primary outcome measure for treatment efficacy In the LEAD study was the absolute intraindividual change of the MADRS total score between baseline and week 8.

### Statistical methods

The study was performed and analyzed using an estimands framework. The presentation of the single-item results is based on the primary estimand that followed a treatment policy strategy according to which intercurrent events (prohibited concomitant treatment, treatment non-compliance, premature treatment discontinuation without subsequent prohibited antidepressant therapy) were ignored and the outcomes data were analyzed as observed. For subjects who switched to alternative antidepressant therapy after prematurely discontinuing treatment, a hypothetical strategy was applied (as if alternative antidepressive treatments were not available) and outcomes data obtained after the switch were replaced using information from placebo-treated patients. This framework was consistent with the requirements of the revised European Medicines Agency Guideline on Clinical Investigation of Medicinal Products in the Treatment of Depression^33^ which came into effect in September 2025.

Change from baseline was tested using analysis of covariance (ANCOVA) with factors for treatment and site and the baseline value as a covariate. Treatment effect estimates for the comparisons of silexan and sertraline with placebo were obtained in separate ANCOVA models, and hence the adjusted mean value and associated variance estimates for the placebo group in both comparisons are numerically not identical even though the same subjects were analyzed. Missing or ignored data were multiply imputed using a pattern mixture model under a missing not at random assumption and using only data of the placebo group. The results presented below apply to the estimand in the full analysis set which included all patients who were randomized and treated. All analyses applying to the analysis of individual MADRS items were exploratory and did not involve formal, confirmatory hypothesis testing. Accordingly, *P*-values are interpreted descriptively and hypothesis-generating rather than hypothesis-testing, as indicators for the statistical importance of an observed effect that might deserve further attention, and treatment group differences associated with a two-sided *P*-value ≤.05 are considered statistically significant in an exploratory sense without adjustment for multiplicity.^34^^,^^35^

## Results

### Participant flow and baseline data

**Figure 1:**
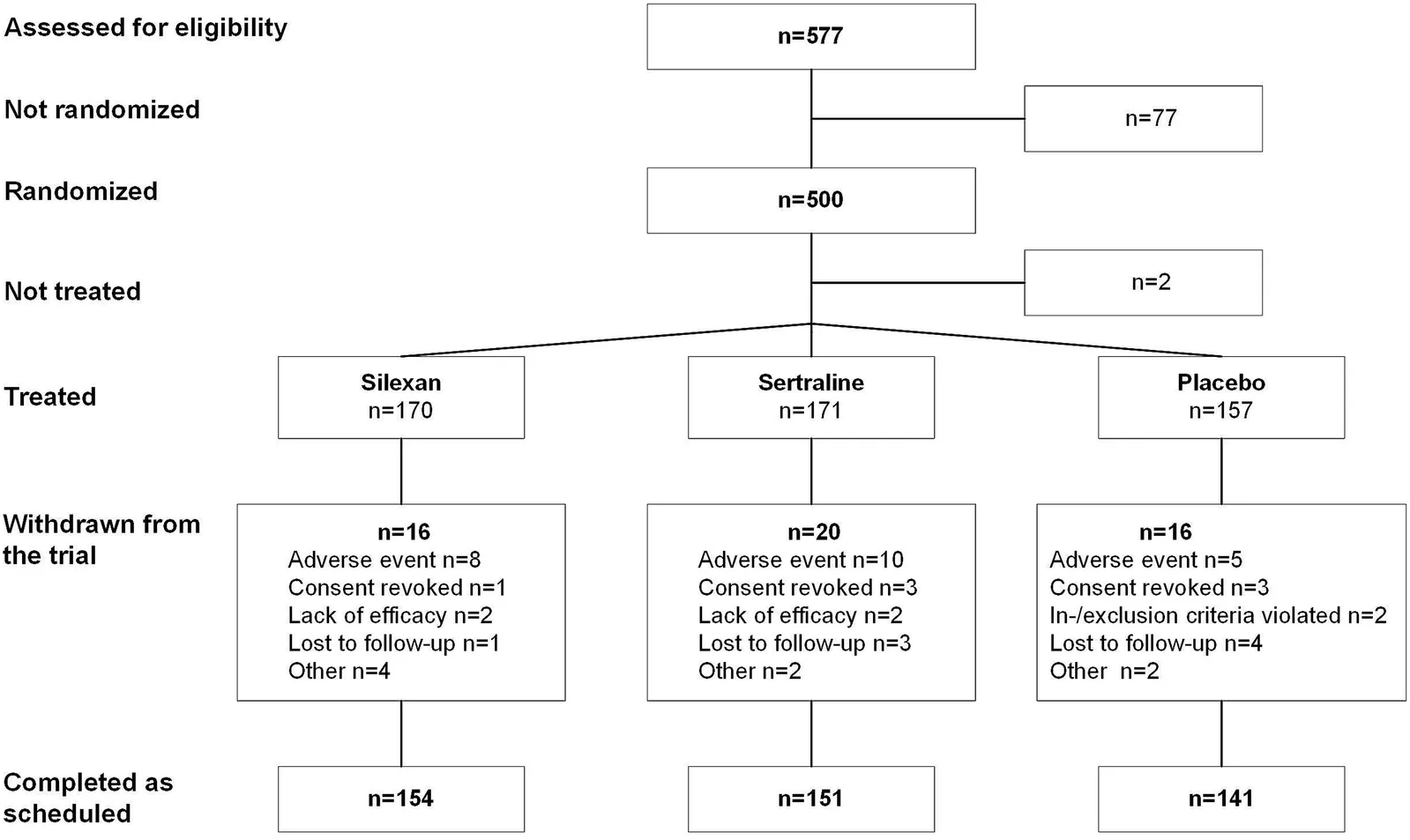
Disposition of subjects, analysis data sets (from Kasper et al.^19^).

A total of 577 subjects were screened in 53 participating sites in Germany and Poland, 500 were randomized, 498 were treated, and 446 (89.2% of 500) completed the study as scheduled (Figure 1). Basic demographic and baseline characteristics of the full analysis set are shown in Table 1. Randomized participants were between 18 and 84 years of age, and about ^2^/_3_ were female. All subjects except five were Caucasians. According to ICD-10 criteria, 230 subjects (46.2%) suffered from mild and 268 (53.8%) from moderate MDD. 216 participants (43.4%) were treated for their first depressive episode while 282 (56.6%) had a recurrent episode. Further sample characteristics are reported in Kasper et al.^19^

**Table 1:** Demographic data and Montgomery-Åsberg Depression Rating Scale at baseline (full analysis set; absolute frequency and % or mean ± SD).

		Silexan (n = 170)	Sertraline (n = 171)	Placebo (n = 157)
**Sex**	Female Male	118 (69.4%) 52 (30.6%)	110 (64.3%) 61 (35.7%)	99 (63.1%) 58 (36.9%)
**Age (years)**		45.8 ± 14.5	44.7 ± 14.4	46.6 ± 15.7
**Months since onset of current episode**		3.3 ± 2.8	3.5 ± 2.9	3.4 ± 2.7
**MADRS – total score and item scores**	Total Score	23.3 ± 3.5	23.6 ± 3.4	23.4 ± 3.4
	1. Apparent Sadness	2.8 ± 1.1	2.6 ± 1.0	2.7 ± 1.0
	2. Reported Sadness	3.1 ± 1.0	3.0 ± 1.0	3.0 ± 1.0
	3. Inner Tension	2.9 ± 0.9	3.1 ± 0.9	2.9 ± 0.9
	4. Reduced Sleep	3.1 ± 1.4	3.2 ± 1.4	3.2 ± 1.4
	5. Reduced Appetite	1.3 ± 1.4	1.1 ± 1.2	1.2 ± 1.5
	6. Concentration Difficulties	2.9 ± 1.0	3.1 ± 1.1	3.0 ± 1.0
	7. Lassitude	2.7 ± 1.1	2.7 ± 1.1	2.8 ± 1.0
	8. Inability to Feel	2.4 ± 1.2	2.6 ± 1.1	2.4 ± 1.2
	9. Pessimistic Thoughts	2.1 ± 1.3	2.1 ± 1.3	2.2 ± 1.3

Abbreviation: MADRS, Montgomery-Åsberg Depression Rating Scale.

Basic demographic and medical history data as well as the total score and the individual item scores of the MADRS were comparable across all treatment groups (Table 1).

### Brief summary of main results

Between baseline and week 8, the MADRS total score decreased by 12.1 (95% confidence interval, CI: 11.0; 13.3) points for silexan, by 12.6 (CI: 11.5; 13.7) points for sertraline and by 10.0 (CI 8.8; 11.1 for comparison vs. silexan, CI 8.9; 11.2 for comparison vs. sertraline) points for placebo (adjusted mean values from ANCOVA models, primary estimand, full analysis set). Both silexan (*P* = .008) and sertraline (*P* = .001) were thus superior to placebo. The proportion of subjects whose MADRS total scores were reduced by at least 50% compared to baseline were 53.5%, 54.0%, and 41.5% for silexan, sertraline, and placebo, respectively. MADRS results were supported by the results of other depression scales as well as by general clinical and functional impairment measures assessed as secondary endpoints (see Kasper et al.^19^ for details and numerical results).

### Analysis of MADRS items

**Figure 2:**
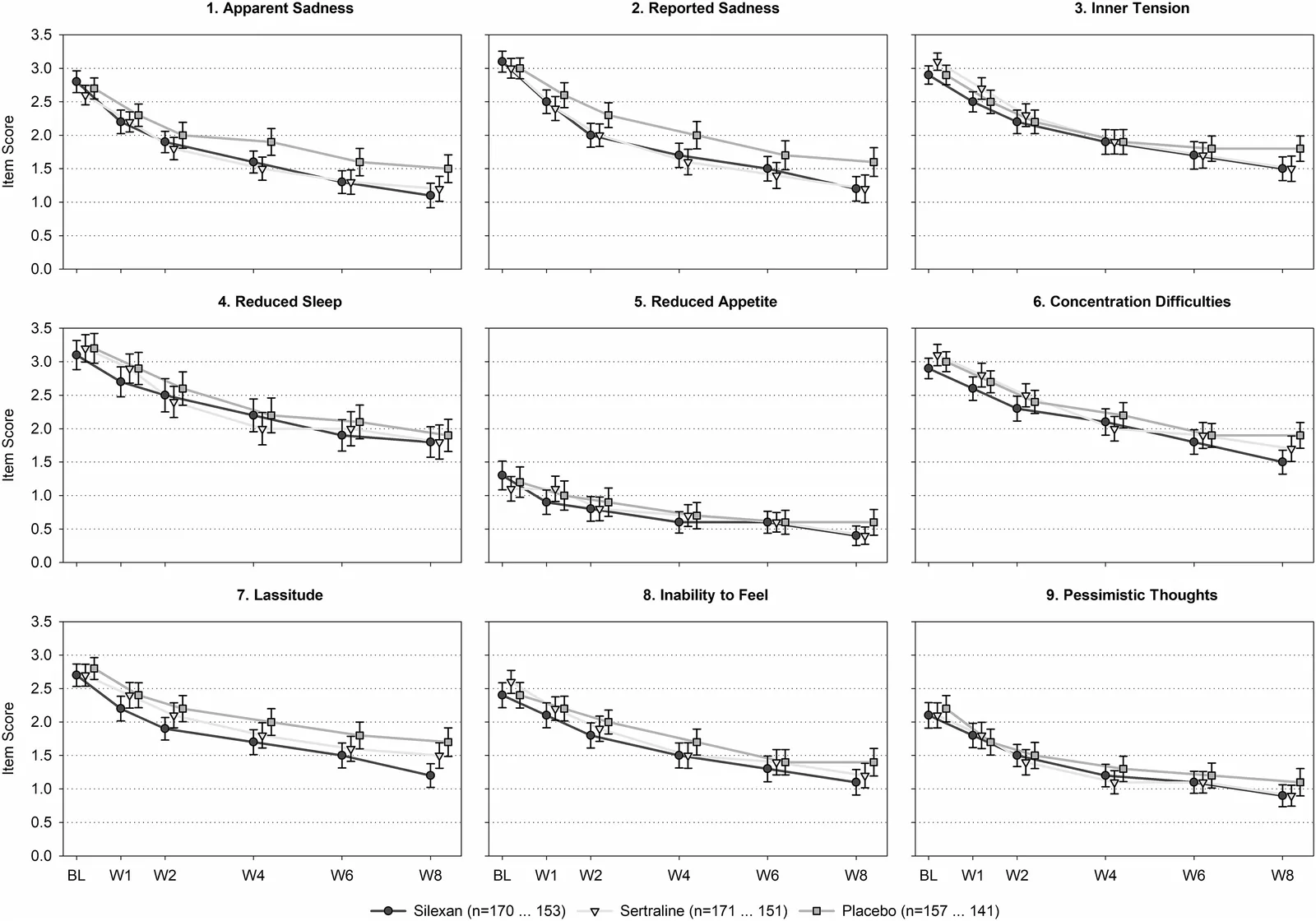
Montgomery-Åsberg depression rating scale individual item scores over time (means and 95% confidence intervals; primary estimand without imputation of missing values, full analysis set; legend shows numbers of subjects with valid data at baseline and week 8; BL – Baseline; W1 … W8 – week 1 … week 8).

At baseline, the observed MADRS item scores were essentially comparable between the treatment groups, with mean value differences between any two groups not exceeding 0.2 points for any item (Table 1). Between baseline and week 8, the average scores of MADRS items 1–9 decreased monotonically in all treatment groups, with consistently more pronounced score decreases in patients treated with silexan or sertraline as compared to placebo (Figure 2). Trajectories indicated an almost exponential decline, with most items showing the largest score decreases per unit of time during the initial 2 weeks of randomized treatment. In the longitudinal perspective, the most consistent advantages of silexan and sertraline over placebo were observed for “apparent sadness” and “reported sadness” as well as for “lassitude”. For “reduced appetite”, average improvements were moderate but consistent, resulting in descriptively significant superiority over placebo in the silexan group despite of a limited potential for improvement in the study sample, with treatment group mean values at or below 1.3 points already at baseline.

**Figure 3:**
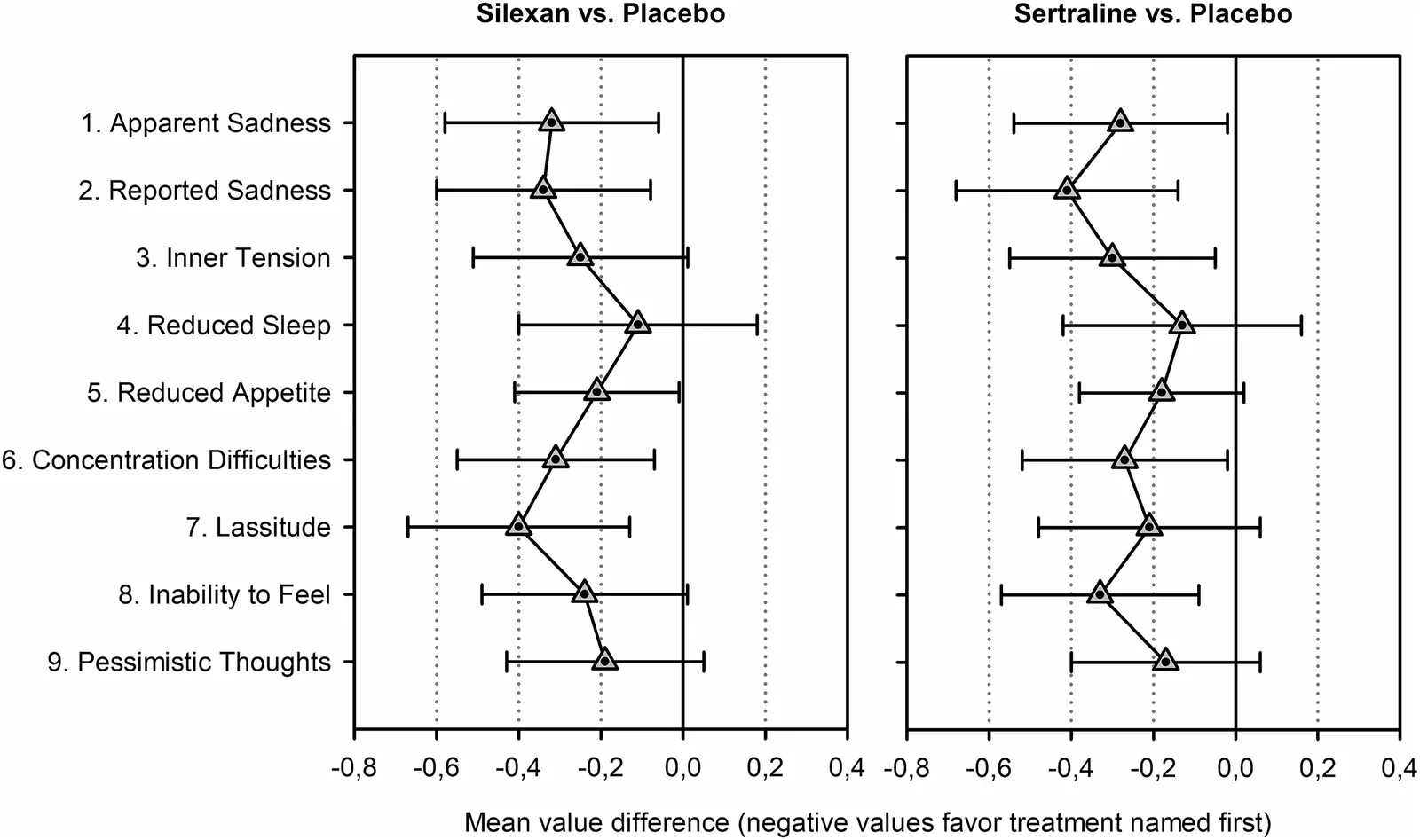
Montgomery-Åsberg depression rating scale individual item scores – Treatment group differences for absolute change between baseline and week 8 (adjusted analysis of covariance mean values and 95% confidence intervals; primary estimand, full analysis set).

For MADRS items 1–9, Table 2 shows the adjusted mean value changes between baseline and week 8 and Figure 3 presents the adjusted means and 95% CIs for the pairwise comparisons between the treatment groups for the same outcomes. Both silexan and sertraline showed exploratory superiority over placebo for five of the nine items; for silexan vs. placebo, two further items showed borderline-significant results (*P* < .06). The largest effect sizes favoring silexan over placebo were observed for “lassitude”, “concentration difficulties”, as well as “reported sadness” and “apparent sadness”, while the largest effects favoring sertraline over placebo were shown for the items “reported sadness”, “inability to feel”, “inner tension”, and “concentration difficulties”. Figure 3 also shows that the placebo-adjusted MADRS item profiles observed for silexan and sertraline were very similar, with comparatively pronounced treatment effects on mood, exhaustion, and concentration, and less pronounced effects on sleep (despite reduced inner tension) and pessimistic thoughts.

**Table 2:** Montgomery-Åsberg Depression Rating Scale item scores – adjusted treatment group mean value differences with 95% confidence intervals for change from baseline to week 8 (primary estimand, full analysis set).

Item	Silexan vs. Placebo			Sertraline vs. Placebo		
	Silexan	Placebo	*P*	Sertraline	Placebo	*P*
**1. Apparent Sadness**	–1.56 (–1.75; –1.37)	–1.24 (–1.43; –1.04)	.014	–1.43 (–1.62; –1.24)	–1.15 (–1.34; –0.96)	.037
**2. Reported Sadness**	–1.83 (–2.02; –1.64)	–1.49 (–1.69; –1.29)	.011	–1.88 (–2.07; –1.69)	–1.48 (–1.68; –1.28)	.003
**3. Inner Tension**	–1.40 (–1.58; –1.22)	–1.15 (–1.34; –0.97)	.051	–1.56 (–1.74; –1.38)	–1.26 (–1.45; –1.07)	.021
**4. Reduced Sleep**	–1.39 (–1.60; –1.18)	–1.28 (–1.49; –1.06)	.447	–1.46 (–1.68; –1.25)	–1.33 (–1.55; –1.11)	.373
**5. Reduced Appetite**	–0.75 (–0.90; –0.60)	–0.54 (–0.70; –0.39)	.048	–0.69 (–0.83; –0.55)	–0.51 (–0.66; –0.37)	.076
**6. Concentration Difficulties**	–1.40 (–1.57; –1.22)	–1.08 (–1.26; –0.91)	.011	–1.41 (–1.59; –1.23)	–1.14 (–1.32; –0.96)	.033
**7. Lassitude**	–1.46 (–1.65; –1.27)	–1.06 (–1.26; –0.87)	.003	–1.26 (–1.45; –1.06)	–1.05 (–1.25; –0.85)	.136
**8. Inability to Feel**	–1.25 (–1.44; –1.07)	–1.01 (–1.20; –0.82)	.058	–1.41 (–1.59; –1.24)	–1.08 (–1.26; –0.90)	.008
**9. Pessimistic Thoughts**	–1.24 (–1.42; –1.07)	–1.05 (–1.23; –0.87)	.118	–1.20 (–1.37; –1.04)	–1.03 (–1.20; –0.86)	.145

For MADRS item 10 (“suicidal thoughts”), one patient in each group had a non-zero score at post-baseline and was subsequently withdrawn from the trial as required by the study protocol.

## Discussion

The LEAD study^19^ demonstrated that the 1 × 80 mg/d dosage of silexan registered for anxiety disorders has a statistically significant and clinically relevant antidepressant effect in patients with mild or moderate MDD. Our analysis complements these results by also demonstrating the effect of silexan at the MADRS item level. At the study population level, all nine of the investigated items indicated more pronounced symptom improvement after 8 weeks of treatment with silexan than was seen in the placebo group. In the exploratory setting of this investigation, five items (“apparent sadness”, “reported sadness”, “reduced appetite”, “concentration difficulties”, “lassitude”) showed statistically important advantages, and two items (“inner tension”, “inability to feel”) had borderline significant results (*P* < .06) favoring silexan. It should be noted in this context that even though the MADRS item-level analysis had been pre-planned, the concept of these analyses was non-confirmatory, and the LEAD study was neither intended nor powered for demonstrating statistical superiority of silexan over placebo on the item level.

The item-level analysis of the MADRS provides further insights into the effect of silexan on specific symptoms of MDD. A comparison of the placebo-adjusted MADRS item profiles for silexan and sertraline (Figure 3) showed essentially comparable effects of the products on the symptoms of MDD. Advantages for sertraline over placebo were in a similar range to those of silexan over placebo. The data therefore suggest that in MDD, sertraline (and possibly also other SSRIs) and silexan may share a similar therapeutic profile with respect to favorable antidepressant and anxiolytic properties but without sedative or sleep-inducing effects.^36–38^ Resemblance between the clinical profiles of silexan and SSRIs has also been observed in animal models^27^ even though silexan is not effective at the classical targets of most antidepressant drugs like monoamine transporters or neurotransmitter receptors and also has no GABAergic properties.^25^^,^^27^ On the other hand, increases of extracellular serotonin and dopamine after administration of silexan have been observed in animal models,^29^ and an imaging in healthy volunteers study found increases the density of 5-HT1A receptors in human brain after subchronic treatment.^28^ Both observations are typical for serotonergic anxiolytics and antidepressants.^27^

Similarities between the MADRS item profiles of silexan and sertraline include the placebo-adjusted change scores for items associated with anxiety and vegetative symptoms, notably “inner tension”, “reduced sleep”, “reduced appetite”, and “concentration difficulties” that were found to load high on a common factor in an exploratory factor analysis of the MADRS.^39^ Changes in anxiety-related items were, however, not more pronounced than changes observed for other items of the MADRS (Figure 3). It thus appears that the antidepressant effect of silexan in MDD, similar to that of sertraline, is not mainly driven by the anxiolytic effect of the product but also by specific antidepressant properties.

Overall, the observed effects of the established and extensively investigated antidepressant drug sertraline underline the assay sensitivity of the trial and thus also strengthen the validity of the results for silexan.

For the MADRS total score, a mean treatment group difference of two points was considered the threshold for the clinically important difference (MCID^40^^,^^41^). Unfortunately, no definition of clinically meaningful between-group differences is available at the item level. However, extrapolating from the MCID defined for the total score, a mean value difference by 0.2 points for single items might be accepted as clinically relevant for purely illustrative and heuristic purposes even though it is acknowledged that this cut-off has not been validated as an MCID in the item level. For the comparison between silexan and placebo, this criterion was met for all items except “pessimistic thoughts”, for which the 0.2-point margin was only narrowly missed (difference: 0.19 points), and “reduced sleep” (Figure 3). It should be noted that the 0.2-point margin was also not reached for these items in the sertraline group either. As regards reduced sleep, a significant and clinically meaningful beneficial effect of silexan was demonstrated earlier in patients with anxiety disorders,^36^ including a significant increase in sleep time based on the Pittsburgh Sleep Quality Index.^42^ A mediation analysis has demonstrated that the beneficial effect of silexan on sleep in the treatment of anxiety disorders is a consequence of the drug’s anxiolytic effect rather than a direct effect, and occurs with a delay.^43^ It is, however, unclear whether clinically meaningful improvements of sleep during treatment with silexan can also be expected in patients with MDD. The item-level data of this trial neither clearly support this nor do they indicate any adverse effects on sleep.

The item-level response offers strong support for a balanced antidepressant effect of silexan. Of note, beneficial effects were observed for items distributed over three out of the four factors (“sadness”, “neurovegetative symptoms”, “detachment”) underlying the MADRS that were identified through factor analysis.^9–11^ The factors “sadness” and “detachment” in particular include those MADRS items focusing on depressed mood, anhedonia, and loss of energy, which are the cardinal symptoms of MDD. The fourth factor, “negative thoughts”, is a summary of MADRS items no. 9 (“pessimistic thoughts”) and 10 (“suicidal thoughts”), the latter of which was not used in our analysis, because participants with an item score of >0 at screening and baseline were excluded from the trial. Therefore, an interpretation of this factor is not considered meaningful.

General strengths and weaknesses of the LEAD study have been discussed previously.^19^ The present item-level analysis provides valuable and detailed additional insights into the therapeutic profiles of the investigated antidepressants and may help clinicians and patients to better understand treatment impact on the symptoms of depression. Item-level analyses of the MADRS have already been published^12^^,^^44^; however, a validated approach to assessing the clinical importance of the observed changes and effects at the item-level, taking into account that different items may have a different MCID, is still lacking. Of course, this also includes the cut-off used in our analyses for the sake of illustration, the validity of which has yet to be demonstrated. The exclusion of patients with non-zero baseline scores for suicidality could be a certain limitation with regard to the external validity of the results even though in mild to moderate depression (the study indication) suicidality is not very common.^45^ It is also important to note that despite their pre-specification, the analyses performed on the single items of the MADRS and the *P*-values computed for them were purely exploratory and should be interpreted in a heuristic context.

In conclusion, this item-level analysis of the MADRS demonstrates a well-balanced antidepressant effect of silexan and thereby enhances the understanding of the changes in the total score. The analysis shows that the most pronounced improvements were observed for the items representing the cardinal symptoms of depression, indicating that silexan may be a treatment choice suitable for patients with various disease patterns.

## Supplementary Material

Three_bullet_points_pyag041

## Data Availability

The data underlying this article were provided by Dr. Willmar Schwabe GmbH & Co. KG, Karlsruhe, Germany, by permission. Raw data cannot be shared both due to ethical reasons and to data protection laws. To the extent permitted by law, the trial data required for validation purposes will be disclosed in result reports on corresponding databases. All relevant data are within the paper.
