# Supplementary material for: Effects of lavender oil preparation silexan on different symptoms of major depression – results from a randomized, controlled trial
Source: Int J Neuropsychopharmacol. 2026 Aug 4;29(9):pyag041. doi: 10.1093/ijnp/pyag041 (PMC13533546; doi:10.1093/ijnp/pyag041)
Supplement: Supplementary file 1 [file three_bullet_points_pyag041.docx]

## Significant outcomes

- In patients with mild to moderate major depressive disorder, lavender oil preparation silexan has a clinical profile similar to that of the selective serotonin re-uptake inhibitor sertraline based on an item-level analysis of the Montgomery-Åsberg Depression Rating Scale (MADRS).
- Silexan has a significant antidepressant effect that includes an alleviation of depressed mood, anhedonia, and loss of energy, the cardinal symptoms of depression. The broad improvement of symptoms of depression could not be explained by the proven anxiolytic efficacy silexan alone but indicates an independent, direct antidepressant effect.
- The present item-level analysis provides valuable and detailed additional insights into the therapeutic profiles of silexan and sertraline and may help clinicians to tailor antidepressant treatment to the specific symptoms of a patient.
